# Tissue Expansion: Further Attempts to Improve Results in Breast Reconstruction

**DOI:** 10.1155/2011/952197

**Published:** 2011-05-10

**Authors:** Donald A. Hudson, Kevin G. Adams, Saleigh Adams

**Affiliations:** ^1^Department of Plastic and Reconstructive Surgery, University of Cape Town Private Hospital and Christiaan Barnard Memorial Hospital, Academy of Plastic Surgery, 310 Library Square, Wilderness Road, Claremont, Cape Town, South Africa; ^2^Department of Plastic Surgery, H53, O.M.B., Groote Schuur Hospital, Observatory, Cape Town 7925, South Africa

## Abstract

Tissue expansion, is a simple method of breast reconstruction. *Method*. A prospective study of 27 patients treated over a 43 month period is described. At the first stage the expander is inserted in the dual plane, and the medial pectoral nerve is divided. The tissue expander is over-expanded. Second stage: a de-epithelialized vertical triangle is used to aid anterior projection, an inframammary fold is created and a silicone gel prosthesis inserted. Z-plasties are added to the transverse scar. The contralateral breast can be treated or left alone. Complications were recorded and the results were assessed by 4 plastic surgeons using a visual analogue scale. *Results*. 19 patients had expanders inserted at mastectomy (2 bilateral) and 8 underwent delayed reconstruction, with a mean age of 47 years (range 30–65 years). A single prosthesis was inserted in 15 patients (mean size 320 mL) and two prosthesis were stacked in 12 patients (mean volume of 400 mL). The mean delay from full expansion to the second stage was 10 weeks (range 3 weeks–11 months). A contralateral augmentation was performed in 5 patients, pexy in 10, a reduction in 2 and in 8 patients no procedure was performed. One patient required explantation. The mean visual analogue assessment was 7. *Conclusion*. This technique should be considered enhance the cosmetic results in tissue expansion.

## 1. Introduction

Tissue expansion is still a popular technique of breast reconstruction [1–7]. Advantages include a quick, relatively simple procedure without donor site morbidity. Additionally, the colour and the texture of the reconstructed breast skin is identical to the contralateral breast [1–8]. It is often considered a two-stage procedure [1–8].

Tissue expanders can be placed electively, as part of a delayed breast reconstruction, or more recently, at the time of nonskin sparing mastectomy. As the mastectomy removes both the nipple-areola complex and breast skin, this skin envelope is reduced in surface area. Tissue expansion allows the skin envelope to be recreated.

Historically, tissue expanders were placed in a total submuscle pocket [8]. However, the expander (and subsequent prosthesis) was often displaced upward by muscle action, leading to an asymmetrical reconstruction. More recently, the expander has been placed in a “dualplane” with improved aesthetic results [1, 9–11].

This paper concerns 27 patients undergoing two-stage tissue expansion over a 43-month period. A number of manoeuvres have been performed in an endeavour to enhance the cosmetic result.

## 2. Material and Methods

All patients having breast reconstruction with tissue expanders between November 2002 and 31st May 2006 were entered into this prospective study.

The age of the patient, size of the tissue expander, and delay between full expansion and removal of the expander, and insertion of the prosthesis were recorded. Postoperative complications for the first stage were recorded. The size of the prosthesis used, and whether the contralateral breast underwent surgery was, recorded. Again, postoperative complications were recorded.

The photographic results of all patients were assessed by a panel of plastic surgeons using visual analogue scale (10 = excellent) (0 = very poor).

## 3. Technique

### 3.1. First Stage, Insertion of Tissue Expander

If the expander is placed as a delayed breast reconstruction, the skin incision used is via the previous mastectomy scar. If placed at time of mastectomy, the skin incision has been designed by the oncological surgeon. In most cases, it is that of a large ellipse (which incorporates the NAC). 

After haemostasis is secured, the inferior insertion of pectoralis major is detached, and this is extended medially.

The medial pectoral nerve emanating through the pectoralis minor is sectioned, but the lateral pectoral nerve is left intact.

The tissue expander is then inserted. It is noted that the lower half or inferior part is subcutaneous and the upper (superior) half is submuscle (dual plane). 

The inferior border of pectoralis is sutured to the inferior flap used for the mastectomy to ensure 2 layer closure of the wounds.

The fascia of the breast (again with 3/0 Vicryl) is closed, and then, the skin is closed with subcutaneous sutures. A suction drain is always placed.

15% of the tissue expander recommended volume is inserted, and after 10 days–2 weeks, regular weekly expansion is undertaken.

Overexpansion is frequently performed to the extent that the size of the expanded breast is at least 1.5–2 times that of the contralateral breast. If an augmentation of the contralateral breast is planned, further overexpansion is performed. The manufacturers recommended volume of the tissue expander is ignored.

### 3.2. Second Stage

This is only undertaken after full expansion has been completed and a period has elapsed such that the overlying skin is soft, supple, and mobile and can be easily “pinched” between 2 fingers.

**Figure 1:**
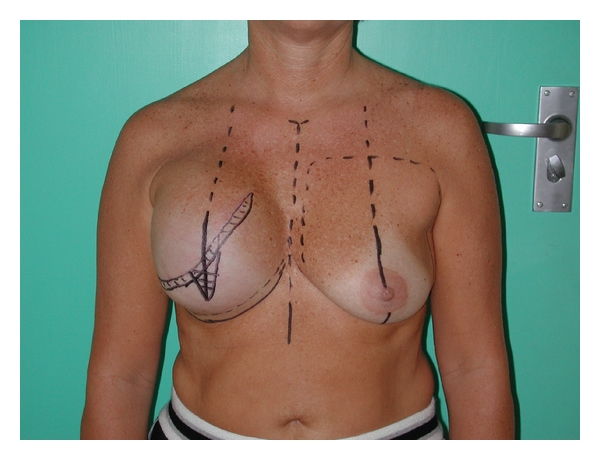
Patient marked prior to second stage. Both a horizontal and vertical de-epithelial area.

Prior to surgery, the patient is marked in a standing position (Figure 1).

There are 3 components to the marking.

**Figure 2:**
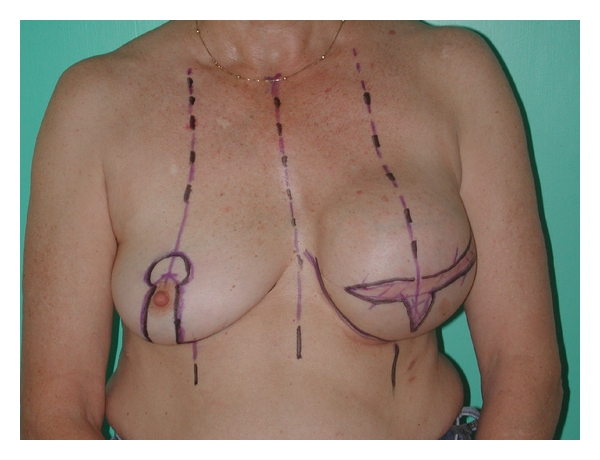
Another patient (slightly oblique view) showing de-epithelialized areas and the two Z-plasties marked on left breast.

- A horizontal de-epithelialized flap is marked below the scar to enable two layer skin closure (Figures 1 and 2).
- A vertical area is marked and will be de-epithelialized. This is designed to assist in achieving projection (Figures 1 and 2).
- The new inframammary fold is marked, which corresponds to the level of the contralateral inframammary fold. A line 1 cm distal (caudal) to this is marked. This is the level where the sutures to create the IMF will be inserted (Figures 1 and 2).

The operation is performed under general anaesthetic, with the patient supine. 

De-epithelialization of the marked areas is done first (Figures 1 and 2).

The previous incision, which had been used for the mastectomy, is opened. The pectoralis major is dissected and detached from the inferior mastectomy flap, and the tissue expander is removed.

A capsulotomy is performed at the base of the capsule circumferentially. The dissection is extended inferiorly in a deep subcutaneous plane to just below the level of the new inframammary fold. 

After haemostasis is secured, the new inframammary fold (IMF) is created.

This is done by taking big horizontal “bites” of the superficial fascia of the abdomen with a 1/0 PDS or 1/0 Nylon suture, 1 cm caudal to the marked inframammary fold, and then suturing this to a rib periosteum.

Multiple sutures (usually about 6–8) are inserted. The loops of sutures are held with an artery (mosquito) forceps, and only once, all the sutures have been inserted, then the sutures are tied.

A second layer of sutures are inserted, but this time, the sutures are orientated in a horizontal direction. The suture passes close to the dermis and is again secured to rib periosteum. 

Only round textured silicone gel prosthesis are inserted. Where the contralateral breast is augmented, or has good projection, 2 silicone gel prostheses are inserted and stacked. 

A suction drain is inserted.

**Closure**

The pectoralis muscle is again sutured to the inferior mastectomy flap with 3/0 Vicryl.

The de-epithelialized vertical segment is closed with horizontal sutures of 3/0 Vicryl.

Then, the de-epithelialized horizontal segment on the inferior mastectomy flap is sutured to the superior flap with 3/0 Vicryl.

Finally, the skin is closed with interrupted 3/0 Vicryl sutures. However, prior to completing closure, 2 Z-plasties are performed at approximately one third and two thirds from the medial edge of the scar.

Strapping is placed to immobilize the prosthesis and prevent both superior and lateral migration.

**Contralateral Procedure**

Patients may also require a simultaneous contralateral procedure, depending on the size and degree of ptosis of the contralateral breast.

## 4. Results

There were 27 patients with a mean age of 47 years (range 30–65) who underwent this two-stage procedure.

In 19 patients, the tissue expander was inserted at the time of mastectomy and in 8 patients, the expander was inserted as a delayed procedure.

Only 1 patient had had radiotherapy following mastectomy, before the expander was inserted, but 2 patients required postoperative radiotherapy after insertion of the tissue expander. Two patients had this procedure performed bilaterally. The most commonly used size of tissue expander was 600 mL (range of 300–600 mL).

Over expansion of the tissue expander beyond the recommended volume was performed in 16 patients with the greatest overexpansion occurring to a volume of 1100 in a patient with a 600 mL tissue expander. In every patient, the final volume of the prosthesis, (even when stacked) was at least 100 mL less than the volume achieved by overexpansion of the tissue expander.

A single gel prosthesis was inserted in 15 patients. The mean volume was 320 mL with a range of 180–540 mL. The prosthesis were stacked in 12 patients. The mean total volume used was 400 mL with a range of 340–550 mL.

The mean time delay from full expansion to the second operative procedure was 10 weeks with a range of 3 weeks–11 months.

**Figure 3:**
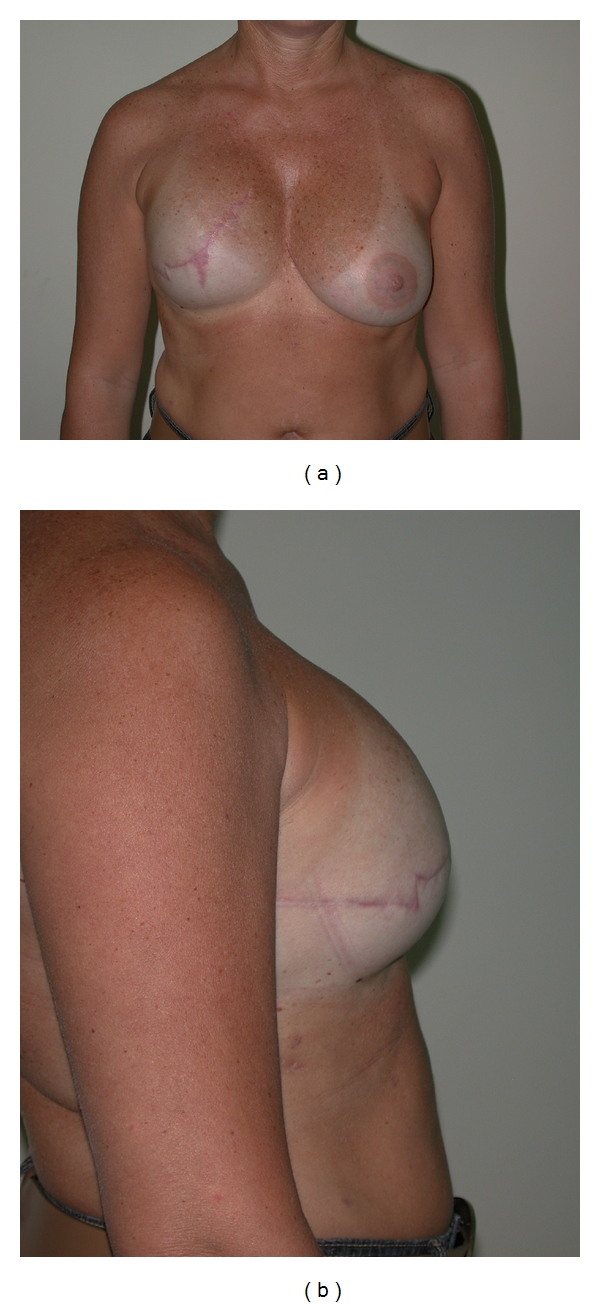
(a) Patient after contralateral augmentation. She scored 9 on visual analogue scale. (b) Lateral view.

**Figure 4:**
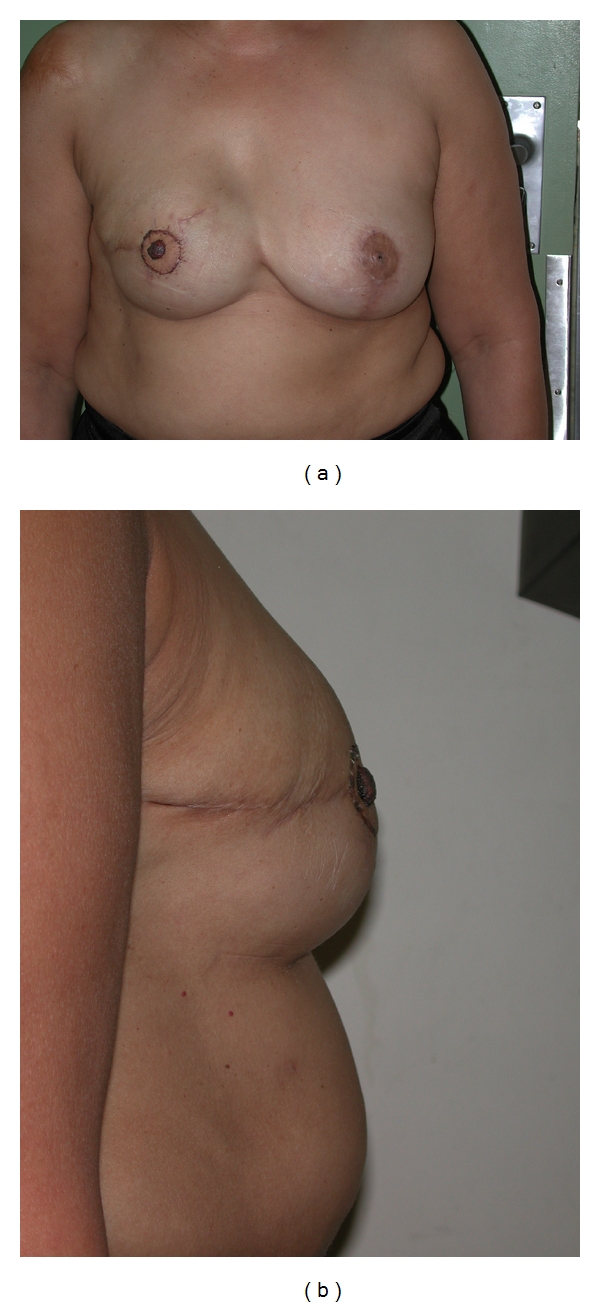
(a) Patient after contralateral breast reduction and nipple-areola reconstruction. She scored 8 on visual analogue scale. (b) Lateral view. Note T-part of scar hidden by NAC reconstruction.

**Figure 5:**
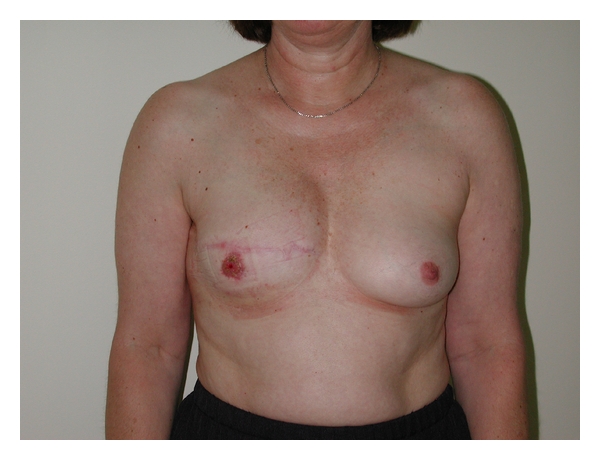
Patient did not have contralateral procedure. She did have failed nipple-areola reconstruction (poor graft take after nipple sharing and skin graft for areola reconstruction). She scored 7 on visual analogue scale.

**Figure 6:**
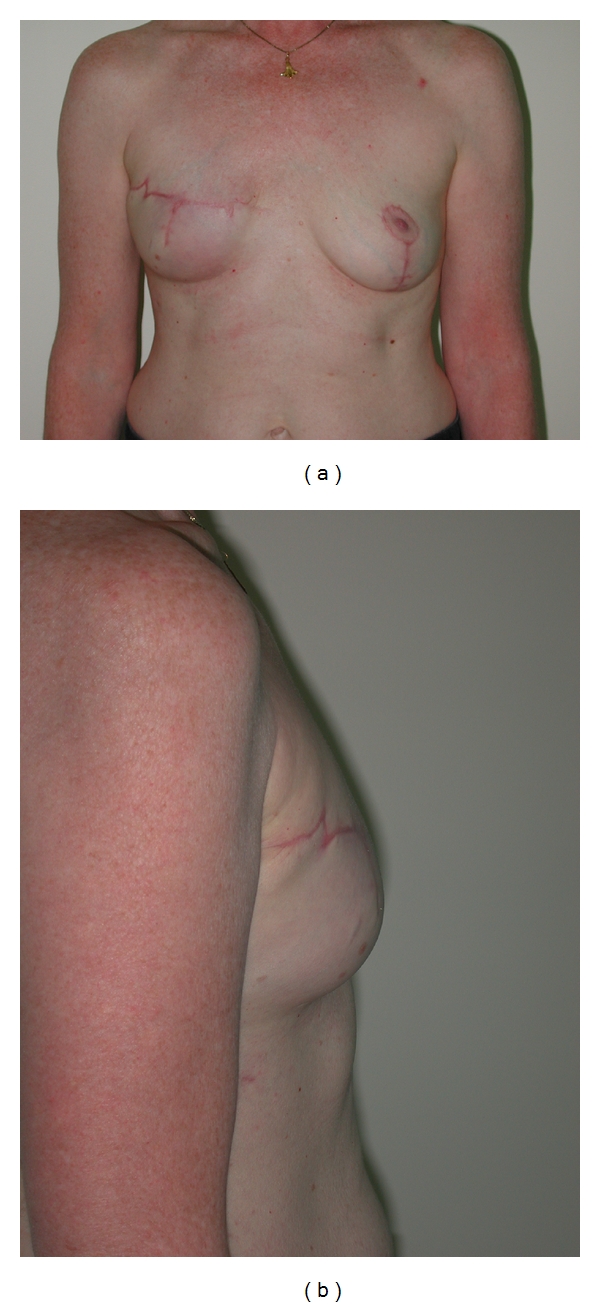
(a) Patient who had contralateral mastopexy. She scored 6 on visual analogue scale. (b) Lateral view.

Two patients had bilateral reconstruction performed. In the other 25 patients, a contralateral augmentation was performed in 5 patients (Figure 3), a contralateral mastopexy in 10 patients (Figure 6), a breast reduction in 2 patients (Figure 4), and in 8 patients, no contralateral procedure was performed (Figure 5).

## 5. Complications

One patient who had prior radiotherapy developed recalcitrant infection, and the prosthesis was removed. Cellulitis occurred in 2 patients and responded to antibiotic therapy. Two patients developed seromas requiring aspiration under ultrasound guidance. 

One patient had dehiscence of the newly created inframammary fold and inferior descent of the prosthesis. One patient had delayed wound healing requiring dressings.

## 6. Followup

The minimum followup is 17 months, with a range of 6–35 months. Three patients have been last to follow up.

## 7. Visual Analogue Scales

The 4 plastic surgeons rated the results with a mean of 7 (range 2–9).

## 8. Discussion

Tissue expansion is still a popular technique of achieving breast reconstruction, whether used immediately at mastectomy or for delayed reconstruction. Previously, the tissue expander was always inserted into a total submuscle pocket because of the fear of skin necrosis, leading to exposure and explantation of the expander. It also provided a second layer of soft-tissue cover for patients who had saline prosthesis inserted [8].

However, the pectoralis major flap adhered to the overlying mastectomy flap. When the pectoralis contracted, it compressed, and thus displaced the tissue expander/ prosthesis superiorly and laterally [12]. This led to asymmetry with little or no ptosis. The normal contralateral breast often required mastopexy to achieve symmetry. The asymmetry was compounded by the lack of definition of the IMF of the reconstructed breast. The inframammary fold had either been destroyed at the mastectomy or, if still present, lacked definition. This is because it is essentially a cutaneous structure [13], which would not be accentuated by a submuscle prosthesis displaced superiorly.

Spear et al. [1, 2] overcame some of these problems by placing the prosthesis in a dual plane. They also applied marionette sutures to the pectoralis major flap in an endeavour to ensure muscle coverage of the upper 2/3 of the prosthesis. However, in our experience of this technique (but not using marionette sutures), the contraction of the pectoralis major still displaced the overlying skin envelope (to which it was attached) superiorly and compressed and displaced the prosthesis superiorly.

A number of manoeuvres are described in an attempt to overcome some of the shortcoming of other techniques.

These are the following.

**(a) First stage (insertion of tissue etxpander)**

(1) Detach the insertion and mobilize pectoralis major muscle [1, 2, 10, 11].

The muscle is detached from its inferior and lower medial insertion until the fifth rib, and the medial pectoral nerve is divided (to lessen the upward traction of the muscle on the mastectomy skin flaps).

The muscle is then sutured to the inferior mastectomy skin flap to provide two-layer closure to the suture line.

This manoeuvre also enables the expansion of the subcutaneous lower pole of the breast envelope to occur without interference from the muscle. The latter allows the creation of breast ptosis, and better symmetry to be achieved (see Figures 3–6).

Disadvantages of this manoeuvre include the fact that the lower third of the prosthesis has less soft tissue coverage and rippling of the prosthesis may be more noticeable although this is less common with a silicone gel prosthesis.

(2) Denervation of part of the pectoralis muscle.

As noted previously, the mobilised pectoralis major flap adheres to thin mastectomy skin flaps, and as the pectoralis major contracts, the skin flaps are retracted superiorly, especially the inferior skin flap. Additionally, these forces tend to displace the prosthesis upwards and laterally. This flattens the breast (particularly noticeable on lateral view) so that the normal breast shape is lost, and there is a loss of the gentle curve from the nipple to the inframammary fold.

Partial denervation overcomes this retractive force, but some muscle tone is retained. It also decreases the lateral displacement of the prosthesis when the patient lifts a heavy weight.

However, care must be taken to ensure that the muscle is not totally denervated to prevent loss of the definition of the anterior axillary fold.

(3) Dual plane.

The dual plan has been alluded to already [1, 2, 7]. This allows the lower pole/subcutaneous portion of the breast to be expanded more easily. It also allows the formation of some breast ptosis, as the skin flaps are not retracted superiorly by the contracting pectoralis muscle.

(4) Overexpansion.

The technique described buries some of the expanded skin, and hence, more skin is simply required. More importantly, skin expansion represents both stretch and new tissue formation [14].

Overexpansion is undertaken to ensure that an adequate pocket can be comfortably achieved without placing tension on the skin flaps. Additionally and importantly, creating ptosis also represents a situation where more skin is required, and this skin must remain lax and not contract.

It appears that tissue expansion works initially by recruitment of (stretch) adjacent tissue. Later, new tissue formation occurs. The period of “waiting” between complete “overexpansion” and the second-stage operation is designed to allow for new tissue formation to occur. Exactly when new tissue formation actually occurs is not known.A clinical decision is undertaken once the skin overlying the expander is soft and supple and can be “pinched” between fingers. The period is at least 1 month but varies between patients, the quality of their skin, and so forth.

It bears repeating that the recommended volume of the tissue expander is ignored. Hallock [15] showed that overexpansion to 10 times the recommended volume can be safely performed. Expansion is continued until the expanded breast is at least 1.5–2 times the contralateral breast.

**(b) Techniques Which Are Undertaken at the Second Stage (i.e., Removal of Tissue Expander and Insertion of the Prosthesis)**

(1) Skin incision.

- The de-epithelialized horizontal segment allows additional protection of the scar. This is important, as the skin flaps may be thin following expansion and indeed overexpansion itself. This technique can, however, only be performed where there is sufficient overexpansion to allow recruitment and burying of “additional tissue”.
- The vertical de-epithelialized triangle is designed to add projection to the breast. This is achieved by adding bulk to the breast by inverting and burying this de-epithelialized triangle.Care must be taken in the planning of the triangle. If the triangle is too wide, the breast mound will be flattened. Alternatively, if too small, little projection will be obtained. Hence, it requires some clinical judgment, and it appears that the triangle at its widest measures about 3–4 cm.The resulting T scar can be partially hidden by subsequent nipple-areola reconstruction (see Figure 4).
- The two Z-plasties are designed to limit the contracture of a big horizontal scar across the meridian of the breast.Most commonly, the mastectomy scar results from a horizontal ellipse and extends from the medial edge of the breast to its lateral edge. However, this scar is not placed in the relaxed skin tissue lines/Langers' lines [16] and, hence, some contraction of the scar occurs [17]. This is sometimes more obvious on the lateral view, as a horizontal band across the fully expanded breast skin with a convexity above and below the scar.

(2) Stacking prosthesis [4]: Stacking is not a new concept. It has previously been applied using smooth walled prosthesis [4]. Stacking in the reconstructive patient allowed better projection to be obtained, particularly in large-breasted patients undergoing mastopexy and in patients undergoing contralateral augmentation.It is important to note that this practise has now been abandoned due to the availability of anatomically shaped implants.

(3) Creation of inframammary fold (IMF): The IMF is one of the critical landmarks of the normal breast and a key factor leading to enhanced results in skin sparing mastectomy is the retention of the inframammary fold [13, 17].Muntan et al. [13] showed that the IMF is formed by the superficial fascia system with attachments to both the skin and deep fascia. Hence, the first row of stitches in creating the IMF attaches superficial fascia to the rib periosteum. The second row of sutures attaches the subdermal tissue to the superficial fascia.Creation of the IMF defines the inferior border of the breast. It is also important in creating a ptotic breast.Disadvantages of this technique are that it is quite painful for the first few days and it is often difficult to get the two folds exactly symmetrical. The IMF sutures dehisced in one patient in this study (a smoker).

## 9. Conclusion

The total mastectomy, unlike the skin sparing mastectomy, leaves a vestige of the original breast skin envelope, and in many cases, the inframammary fold is also destroyed. The breast envelope needs to be reconstructed, the inframammary fold needs to be reconstructed, as well as the breast parenchyma. Additionally, the contralateral breast (which underwent surgery of some sort in two thirds of patients in this study) needs to be assessed for size and shape, and degree of ptosis.

There are a number of other points that also deserve mention. Capsular contracture is not addressed, as there are so many variables that occur in a two-stage procedure. Furthermore, long-term followup is required if this is to be accurate. Also, some of the manoeuvres performed are based on clinical judgment and are difficult to quantify. For example, the degree of overexpansion required is a clinical one. Similarly, the time from completed expansion to the second stage is also based on a clinical assessment that the skin overlying the expander is soft, pliable, and mobile, not taunt and immobile. 

However, despite these limitations, it appears that a satisfactory result can be achieved. Each step is based on careful observation, and the manoeuvres described particularly pectoralis muscle denervation, overexpansion, and a modified skin incision are made in an attempt to improve results. Tissue expansion has many advantages. The cosmetic result should be one of them.
